# The Antinociceptive Effect of Nicorandil in Neuropathic and Nociceptive Pain is Partially Mediated via TRPV1/Opioidergic Signaling

**DOI:** 10.1007/s12035-025-05136-5

**Published:** 2025-06-20

**Authors:** Rasha M. Badr, Salwa A. Abuiessa, Samar S. Elblehi, Nayera W. Hassan, Elham A. Afify

**Affiliations:** 1 Department of Pharmacology and Toxicology, Faculty of Pharmacy, University of Alexandria, 1-El-Khartoum Square, Azarita, Alexandria, Postal Code: 21521 Egypt; 2 Department of Pathology, Faculty of Veterinary Medicine, University of Alexandria, Alexandria, Egypt; 3 Department of Pharmaceutical Chemistry, Faculty of Pharmacy, University of Alexandria, Alexandria, Egypt

**Keywords:** Neuropathic pain, Nicorandil, TRPV1, Opioidergic

## Abstract

Neuropathic pain is a serious neurological disorder caused by lesioned somatosensory neurons characterized by multiple pathologies. Transient receptor potential vanilloid 1 (TRPV1) channels and opioid receptors are co-expressed in dorsal root ganglia (DRG) and play a crucial role in the development of neuropathic pain. Here, we investigated the possible involvement of TRPV1 channels and µ-opioid receptors in mediating the antinociception of the K_ATP_ opener, nicorandil, in neuropathic pain in four nociceptive models: chronic constriction injury of the sciatic nerve (CCI), formalin, capsaicin, and acetic acid writhing tests. Nicorandil (150 mg/kg, twice, 2 h apart, PO) administered to male rats (i) reversed the effects of CCI on nociceptive threshold and cumulative scores assessed by von Frey and acetone test, respectively; (ii) reduced licking time and number of flinches in biphasic formalin and capsaicin tests, and (iii) reduced the number of writhes in the acetic acid test; and (iv) combined nicorandil-capsaicin abolished acetic acid induced writhing response. Similarly, ipsilateral intraplantar injection of nicorandil (37.5 mg/paw, twice, ipl) inhibited nociceptive responses induced by capsaicin, formalin, and acetic acid. Immunohistochemical analysis revealed that nicorandil blunted the CCI-induced elevation of TRPV1 protein expression in DRG. The beneficial effects of nicorandil in all models were attenuated by naloxone. Molecular docking supported the interaction between nicorandil and TRPV1. Histologically, nicorandil improved the pathological changes induced by CCI in the sciatic nerve and DRG. Collectively, these results demonstrate that nicorandil exhibits antinociceptive effects in neuropathic and nociceptive pain via mechanisms involving TRPV1 modulation and opioid receptor signaling. Further investigation is warranted to explore the mechanism of action of nicorandil as an alternative treatment option for neuropathic pain.

## Introduction

Pain is defined according to the International Association for the Study of Pain (IASP) as “unpleasant sensory and emotional experience associated with actual or potential tissue damage” [1]. Neuropathic pain is a debilitating chronic pain that occurs as a consequence of a lesion or a disease of the somatosensory system. The prevalence of neuropathic pain accounts for 7–8% of the general population and 25% of chronic pain [2]. Patients with neuropathic pain suffer from both spontaneous and evoked pain; the latter can be classified into allodynia, which is pain to non-noxious stimuli, and hyperalgesia, which is exaggerated pain to noxious ones [2]. Neuropathic pain may develop peripherally, as in postherpetic neuralgia, trigeminal neuralgia, radiculopathies, painful polyneuropathies, and peripheral nerve injury, or centrally, as in spinal cord injury, brain injury, stroke, and multiple sclerosis, which explains the multiple pathologies and poor therapeutic outcomes of neuropathic pain [3, 4].

Transient receptor potential (TRP) channels, calcium, sodium, and purinergic channels have crucial roles in chronic pain due to their localization in sensory neurons and other pain processing structures [5]. The opening of potassium channels can block transient action potentials by hyperpolarization [6]. Pharmacological targeting of these ion channels is recently being investigated in the management of chronic pain [7].

Transient receptor potential vanilloid subfamily member 1 (TRPV1) is a polymodal channel that can be activated by different stimuli such as high temperature, acidic pH, and chemical compounds, including capsaicin and has a vital role in nociception [8]. The heightened TRPV1 expression is reported in different models of neuropathic pain [9–11]. The pharmacological inhibition of TRPV1 channels relieves neuropathic pain in chronic constriction injury (CCI) rats [10, 12]. Therefore, TRPV1 modulation seems to be an intriguing target to mitigate pain [13].

The pharmacological classes involved in the management of neuropathic pain are tricyclic antidepressants, serotonin-noradrenaline reuptake inhibitors, antiepileptics, opioid analgesics, and capsaicin [4]. However, the use of these classes is associated with inadequate pain relief and multiple side effects [3]. Thus, novel safe and effective drugs are needed to improve patients’ response and relief from neuropathic pain.

Nicorandil is an ATP-dependent potassium channel opener used as a direct vasodilator in angina pectoris [14] and demonstrated improved pain sensation in patients with angina over nitroglycerin [15]. Taking into account that nicorandil is approved only for the treatment of cardiovascular complications, it has been receiving attention for its analgesic potential in different models of pain [16–20]. Nicorandil was found to attenuate experimental post-operative pain in rats, where the pain-relieving activity is suggested to involve the inhibition of P120 expression in the spinal cord and DRG [19]. Recent studies demonstrated its antinociceptive activity in other pathological disorders such as liver fibrosis [21, 22] and arthritis [23]. Research studies showed antinociceptive activity of nicorandil in inflammatory, neuropathic, and post-surgical pain [16–20]. Nicorandil demonstrated antinociceptive and anti-inflammatory effects against different models of experimental pain, including rat formalin and carrageenan models. Its antinociceptive and anti-inflammatory potential is speculated to be mediated through the opioid pathway [18]. In addition, nicorandil is reported to relieve neuropathic pain in paclitaxel and surgery models in rats possibly through serotonergic and opioidergic pathways [20]. However, the full mechanism underlying nicorandil’s analgesic potential is still not well understood.

Opioid receptor agonists are the gold standard intervention for pain management. Opioid receptors are co-expressed with TRPV1 ion channels in dorsal root ganglia neurons (DRG). The activation of µ-opioid receptor inhibits TRPV1 action in a G-protein-cAMP dependent mechanism. This interaction sheds light on the TRPV1 channels as a possible target underlying the antinociceptive activity of µ-opioid receptor ligands [24]. Therefore, the present study was designed to investigate whether nicorandil exerts antinociceptive effects in both neuropathic and nociceptive pain models and to explore the potential involvement of TRPV1 and µ-opioid receptor signaling in mediating its action. This was addressed in different pain models, including chronic constriction injury of the sciatic nerve (CCI), formalin and capsaicin tests, as well as the acetic acid induced writhing test, and was verified using pharmacological, histological, and immunohistochemical investigations.

## Material and Methods

### Drugs and Reagents

Nicorandil (A gift from Adwia pharmaceuticals®, Egypt) was freshly prepared as a suspension in 0.5% CMC in sterile saline. Naloxone (Sigma Aldrich®) was dissolved in sterile saline (1 mg/ml). Capsaicin (Sigma Aldrich®) was prepared as a stock solution of 3 mg/10 ml in pure ethanol to be diluted before experiments with saline (saline/ethanol, 2:1). Capsazepine (Sigma Aldrich®) was prepared as a stock solution of 5 mg/ml in DMSO to be diluted before experiments with saline (saline/DMSO, 5.25:1). Formalin 1% and acetic acid 0.6% were prepared in saline immediately before experiments.

### Animals

In total, 230 male Wistar rats (180–230 g) were obtained from the animal house of the Faculty of Pharmacy, Alexandria University. Rats were kept at room temperature and 40–60% humidity in a 12 h light/12 h dark cycle with free access to chow (19% protein, AL- Fajr feed Co., Egypt) and water. Rats were randomly divided using a simple randomization sequence into four series according to the pain model, namely, CCI, formalin test, capsaicin test, and acetic acid writhing test. The study was performed according to the institutional recommendations for the Animal Care and Use Committee of the Faculty of Pharmacy, Alexandria University (approval no. AU06201958150).

### Chronic Constriction Injury (CCI) of Sciatic Nerve

Rats were anesthetized using thiopental sodium (50 mg/kg, I.P). The common sciatic nerve was exposed by blunt dissection through the biceps femoris muscle at the middle of the thigh. The nerve was liberated from adhering tissue. Then, four ligatures (silk, 4/0) were tied loosely around the nerve, with 1-mm spacing from each other proximal to the trifurcation of the sciatic nerve. In sham-operated rats, the same surgical steps were performed without nerve ligation. This model is widely conducted to study neuropathic pain because it represents a good match for human chronic neuropathic pain [25]. Nicorandil was administered on day 14 post-surgery (150 mg/kg, twice, 2 h apart, PO). The evaluation of mechanical allodynia was performed at 1, 3, 5, and 7 h after the first dose of nicorandil [20]. Cold allodynia was evaluated at 1.5, 3.5, 5.5, and 7.5 h after the first dose of nicorandil.

#### Assessment of Mechanical Allodynia in CCI Rats

To evaluate mechanical allodynia, rats were habituated in plastic cages placed on a metal wire mesh floor (1 h/day for 2 days) and then, the basal paw withdrawal threshold was measured for each rat as the mean of three measurements (with 3 to 5 min interval between successive applications) using an electronic von Frey apparatus (panlab, Harvard, Spain). After surgery, the paw withdrawal threshold was assessed on alternate days to day 14 [20]. A maximal cut-off value of 60 g was used to prevent tissue damage [26].

#### Assessment of Cold Allodynia in CCI Rats

Ten min after von Frey test, cold allodynia was tested by performing acetone test. In the same plastic cages, 50 µl of acetone was sprayed to mid-plantar skin surface causing cooling of the skin to non-nociceptive temperature 15–21 °C. Rats were monitored within 1 min and assessed according to the following scoring criteria: 0, no response; 1, mild paw withdrawal or flicking; 2, prolonged flicking of hind paw or stomping; and 3, repeated flicking with licking hind paw. The test was repeated three times with a 2-min interval, and the cumulative score was calculated [27].

Mechanical and cold allodynia were evaluated before (basal values) and every 2 days during the 14-day observation period after surgery. For comparison among different groups, day14 evaluated measurements were only employed [20, 28]. On day14 after surgery, the evaluation of mechanical allodynia was performed at 1, 3, 5, and 7 h [20] while cold allodynia was evaluated at 1.5, 3.5, 5.5, and 7.5 h after the first dose of nicorandil.

### Formalin Test

Thirty min were allowed for rats’ acclimatization in a transparent acrylic box 46 × 46 × 23 cm before the injection of formalin solution (50 µl, 1%) in the plantar surface of the right hind paw (ipl). Licking time and the number of flinches during phase 1 (0–10 min) and phase 2 (10–60 min) were evaluated [29, 30]. Nicorandil (150 mg/kg, twice, 2 h apart, PO) and (37.5 mg/paw, twice, 2 h apart, ipl) were injected 30 min prior to the formalin injection.

### Capsaicin Test

Thirty min adaptation period was allowed in acrylic box 46 × 46 × 23 cm, before rats were injected with capsaicin (2 µg/20 µl, ipl). The licking time and number of flinches were calculated for 10 min [31, 32]. Nicorandil was injected orally (150 mg/kg, twice, 2 h apart) and ipl (37.5 mg/paw, twice, 2 h apart) 30 min prior to capsaicin injection.

### Acetic Acid Induced Writhing Test (Proton Induced Pain)

After habituation for 30 min in acrylic box 46 × 46 × 23 cm, rats were administered acetic acid (0.6%, 10 ml/kg, I.P). The number of writhes characterized by waves of abdominal muscle contractions accompanied by extension of the hind limb within 30 min was counted [33, 34]. Nicorandil was administered orally (100, 150, and 150 mg/kg, twice, 2 h apart) as well as ipl (37.5 mg/paw, twice, 2 h apart) 30 min before acetic acid injection.

### TRPV1 Expression Level by Immunohistochemistry

The protein expression of TRPV1 channels in rat DRG tissues was assessed. DRG tissues were fixed in 10% formaldehyde and embedded in paraffin blocks. Sections (4 µm thick) of DRG were cut and mounted on positively charged adhesive glass slides (Thermo Scientific, Berlin, Germany), then deparaffinized in xylene and rehydrated in a series of declining ethanol concentrations (100, 95, and 70%). Heat-induced epitope retrieval was carried out by immersing slides in coplin jars containing 10 mM citrate buffer solution and incubated in a microwave at power 100 for 1 min, then power 30 for 9 min. Endogenous peroxidases were blocked by 0.3% hydrogen peroxide for 10 min. The rabbit anti-rat TRPV1 polyclonal antibody (1:200, Thermo Fisher Scientific) was applied to the slides and then sections were incubated at 4 °C overnight. The secondary antibody horseradish peroxidase was applied for 30 min. The chromogen 3,3′-diaminobenzidine was prepared and applied as instructed by the manufacturer for protein visualization. Slides were counterstained with hematoxylin and dipped in ascending concentrations of alcohol and then xylene. Images of DRG tissues were taken by OptikamB9 digital camera (Optika Microscopes, Italy). Fiji Image J Software Version 1.51n (National Institutes of Health, Bethesda, Maryland, USA) was used to measure the percentage of chromogen 3,3′-diaminobenzidine positive stained area in DRG.

### Molecular Docking Procedure

The docking study was conducted using Molecular Operating Environment (MOE, 2019.0102) software (Chemical Computing Group, Montreal, Canada, https://www.chemcomp.com/), according to the docking protocol [35]. The Cryo-EM structure of the human TRPV1 receptor (PDB ID: 8GFA) was downloaded from the Protein Data Bank website and then loaded into the MOE program. After adding explicit hydrogen atoms to the protein, partial charges were computed, then followed by employing the “protonate 3D” function to finish the preparation. A database comprising nicorandil and capsazepine was created and prepared for docking by adding hydrogens, calculating partial charges, and energy minimization using Force Field MMFF94x. The active site was determined using the co-crystallized ligand, and the database was docked using the MOE-Dock. The default MOE’s parameters were applied: Triangle Matcher as the placement method, London dG as the scoring function, and ten retained poses. The rigid receptor method was followed as a refinement step with GBVI/WSA dG scoring function. The output database displayed the energy score between the ligand’s conformers and receptor binding sites, expressed in Kcal/mol. All receptor-ligand complexes were inspected visually to determine the most favorable pose in terms of hydrogen bonding, hydrophobic, and ionic contacts with the binding pocket residues. The docked complex exhibiting the highest docking score and similar binding pattern to the reference ligand (capsazepine) was chosen to depict the protein–ligand interactions. All graphical representations were rendered by MOE, 2019.0102.

### Histopathological Examination

Histopathological examination was performed according to [36]. After excision, the sciatic nerves and DRG (L4-L5) (*n* = 5 each) were fixed in 10% neutral buffered formalin and then embedded in paraffin. Paraffin Sects. (5 µm thickness) were deparaffinized in xylene before staining with hematoxylin and eosin (H&E). Stained sections were blindly evaluated using a light microscope (Leica, DM500) and photographed at a magnification of × 400 using a digital camera (EC3, Leica, Germany) [37].

### Experimental Groups and Protocol

**Fig. 1:**
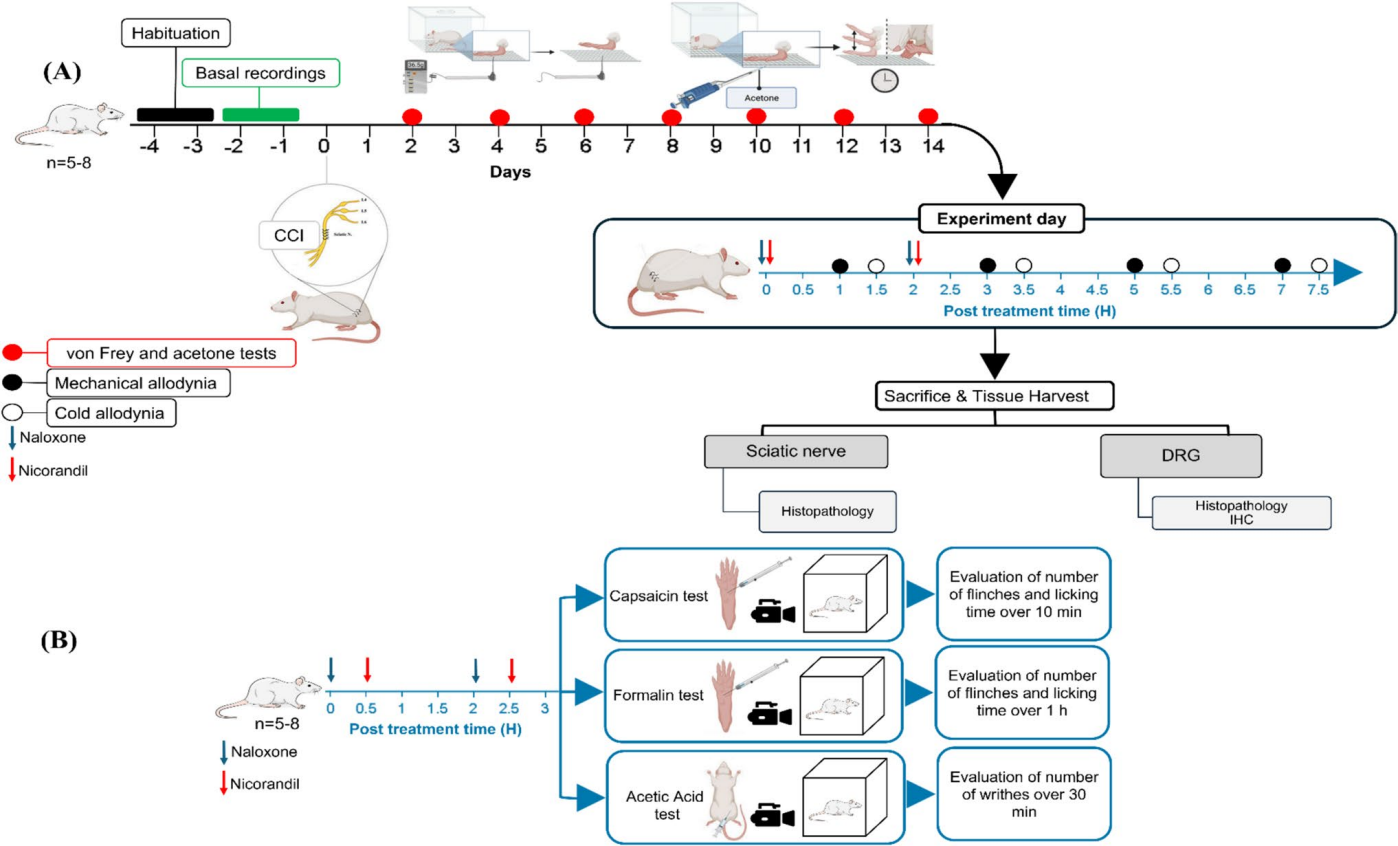
Experimental protocol and time schedule for drug administration in neuropathic pain (**A**) and nociceptive pain models (**B**). IHC, immunohistochemistry; DRG, dorsal root ganglia

The effect of nicorandil was examined alone and in the presence of naloxone in four nociceptive models: namely CCI model, formalin test, capsaicin test, and acetic acid writhing test. Nicorandil was administered orally (150 mg/kg, twice, 2 h interval) [20]. ipl nicorandil (37.5 mg/paw, twice, 2 h apart) was also investigated in capsaicin, formalin, and acetic acid writhing tests. Experimental protocol and time schedule for drug administration are illustrated in Fig. 1.

#### Effect of Systemic Administration of Nicorandil

##### CCI Model

Rats were randomly divided into five groups (*n* = 5–8): one sham group and four CCI groups received treatment as follows: (1) CMC, (2) nicorandil (150 mg/kg, twice, 2 h apart, PO), (3) nicorandil + naloxone (1 mg/kg, twice, 2 h apart, I.P), and (4) naloxone (1 mg/kg, twice, 2 h apart, I.P). Nicorandil was administered on day 14 after surgery. Naloxone was administered simultaneously with each dose of nicorandil. Mechanical allodynia was assessed after 1, 3, 5, and 7 h after the first nicorandil dose. Cold allodynia was assessed at 1.5, 3.5, 5.5, and 7.5 h after the first nicorandil administration. At the end of the experiment, rats were sacrificed using an overdose of thiopental (100 mg/kg, I.P). The ipsilateral sciatic nerves and L4-L5 DRG were isolated for immunohistochemistry and histopathological examination.

##### Formalin Test

Rats were randomly divided into five groups (*n* = 5–8), one received the vehicle, and four groups received formalin and were allocated as follows: (1) CMC (positive control), (2) nicorandil (150 mg/kg, twice, 2 h apart, PO); (3) nicorandil + naloxone (1 mg/kg, twice, 2 h apart, I.P); and (4) naloxone (1 mg/kg, twice, 2 h apart, I.P). The second dose of nicorandil was administered 30 min before the formalin injection. Naloxone was administered 30 min before each dose of nicorandil. The antinociceptive effect of nicorandil was evaluated by measuring the decrease in the number of flinches and the licking time of the rat’s paw.

##### Capsaicin Test

Rats were randomly divided into five groups (*n* = 5–8); one control group received the vehicle of capsaicin (ethanol + saline 1:2, 20 µl/paw, ipl), and four groups were injected with capsaicin (2 µg/paw, ipl) and received the following: (1) vehicle of nicorandil (CMC); (2) nicorandil (150 mg/kg, twice, 2 h apart, PO); (3) nicorandil + naloxone (1 mg/kg, twice, 2 h apart, I.P); and (4) morphine (5 mg/kg, I.P). The second dose of nicorandil was administered 30 min before the capsaicin injection. Naloxone was administered 30 min before each dose of nicorandil. Morphine was administered 30 min before capsaicin. The antinociceptive effect of nicorandil was evaluated by measuring the decrease in the number of flinches and the licking time of paws in rats.

##### Acetic Acid Writhing Model

Rats were divided into eight groups (*n* = 5–8): one negative control group received the vehicle, and seven groups received acetic acid and were allocated as follows: (1) the vehicle of nicorandil (CMC), (2) nicorandil (100 mg/kg, PO), (3) nicorandil (150 mg/kg, PO), (4) nicorandil (150 mg/kg, twice, 2 h apart, PO), (5) nicorandil (100 mg/kg) + naloxone (1 mg/kg, I.P), (6) nicorandil (150 mg/kg) + naloxone (1 mg/kg, I.P), and (7) nicorandil (150 mg/kg, twice, 2 h apart, PO) + naloxone (1 mg/kg, twice, 2 h apart, I.P). The second dose of nicorandil was administered 30 min before the acetic acid injection. Naloxone was administered 30 min before each dose of nicorandil. The antinociceptive effect of nicorandil was evaluated by measuring the decrease in the number of writhes in rats. An additional set of experiments was conducted to verify the interaction of nicorandil with TRPV1 receptors. Rats receiving acetic acid were allocated into four groups as follows: (1) the vehicle of nicorandil (CMC), (2) capsaicin (4 µg/paw, ipl), (3) nicorandil (100 mg/kg, PO) + capsaicin (4 µg/paw, ipl), and (4) nicorandil (150 mg/kg, PO) + capsaicin (4 µg/paw, ipl). The antinociceptive effect of nicorandil was evaluated as mentioned.

#### Effect of Intraplanar Administration of Nicorandil

##### Formalin Test

Rats were divided into five groups (*n* = 5–8), one group received the vehicle and four groups received formalin and were allocated as follows: (1) vehicle of nicorandil (DMSO), (2) nicorandil (37.5 mg/paw, twice, 2 h interval, ipl), (3) capsazepine (16 µg/paw, ipl), and (4) nicorandil (37.5 mg/paw, twice, 2 h interval, ipl) + naloxone (40 µg/paw, twice, 2-h interval, ipl). The second dose of ipl nicorandil was administered 30 min before formalin injection. Naloxone was administered 30 min before both doses of nicorandil. Capsazepine was administered 15 min before formalin. The antinociceptive effect of nicorandil was evaluated by measuring the decrease in the number of flinches and the licking time of paws in rats.

##### Capsaicin Test

Rats were divided into five groups (*n* = 5–8) as follows: One control group received the vehicle of capsaicin (ethanol + saline 1:2, 20 µl/paw, ipl), and four groups injected with capsaicin (2 µg/paw, ipl) received the following: 1) vehicle of nicorandil (DMSO), (2) nicorandil (37.5 mg/paw, twice, 2 h interval, ipl), (3) capsazepine (16 µg/paw, ipl); and (4) nicorandil (37.5 mg/paw, twice, 2 h interval, ipl) + naloxone (40 µg/paw, twice, 2 h interval, ipl). Nicorandil’s second dose was injected 30 min before capsaicin. Capsazepine was administered 15 min before capsaicin. Naloxone was administered 30 min before each dose of nicorandil. The antinociceptive effect of nicorandil was evaluated by measuring the decrease in the number of flinches and licking time of paws in rats.

##### Acetic Acid Induced Writhing Test

Rats were divided into five groups (*n* = 5–8), one group received the vehicle, and four groups received acetic acid and were allocated as follows: (1) vehicle of nicorandil (DMSO), (2) nicorandil (37.5 mg/paw, twice, 2 h interval, ipl), (3) capsazepine (16 µg/paw, ipl), and (4) nicorandil (37.5 mg/paw, twice, 2 h interval, ipl) + naloxone (40 µg/paw, twice, 2 h interval, ipl). Nicorandil’s second dose and capsazepine were injected 30 min before acetic acid. Naloxone was administered 30 min before each dose of nicorandil. The antinociceptive effect of nicorandil was evaluated by measuring the decrease in the number of writhes in rats.

### Statistical Analysis

The number of animals per group was calculated using (G*Power software, version 3.1.9.7) with an alpha of 0.05 and 80% statistical power. Based on pilot studies, Cohen’s d effect size was calculated as 1.2 for nociceptive tests. The total sample size of the 5 groups is 27, and so, the number per group will be a minimum 5 rats. Results were expressed as mean ± standard error of the mean (SEM). Results of von Frey and acetone test were analyzed using unpaired t-test comparing sham and CCI rats during the 14 days after surgery and by two-way ANOVA after treatment on day 14 followed by Tukey’s post hoc test in the temporal course of the mechanical and cold allodynia test and by one-way ANOVA in all other experiments followed by Tukey’s post hoc test. *P*-value < 0.05 was considered statistically significant. Statistical analysis was conducted using GraphPrism software, version 8.0.2. Data were tested for normality using the Shapiro–Wilk test. The Pearson correlation test was used to calculate the correlation between the number of flinches and licking time in the second phase of the formalin test and cold allodynia in CCI rats.

## Results

### Effect of Nicorandil on Mechanical and Cold Allodynia in CCI Rats

**Fig. 2:**
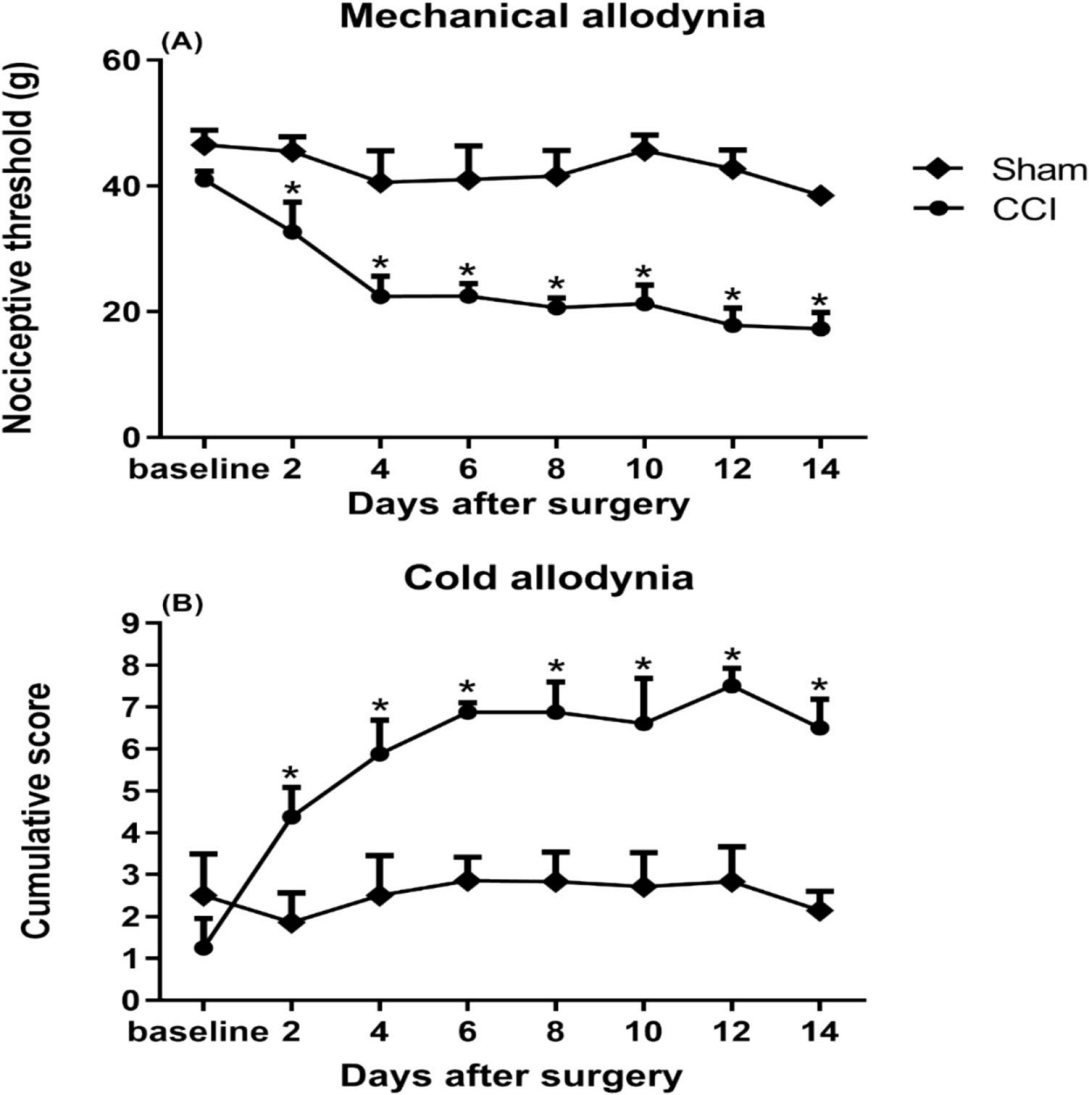
Mechanical (**A**) and cold allodynia (**B**) induced by chronic constriction injury of sciatic nerve (CCI) in male rats. Mechanical nociceptive threshold and cumulative scores were assessed by von Frey and acetone tests, respectively, on alternate days from day 2 after surgery to day 14. All results were expressed as mean ± SEM (*n* = 5–8). Results were analyzed by multiple unpaired *t*-test. *P < 0.05 vs sham

Mechanical and cold allodynia characterized by paw withdrawal threshold and cumulative scores, respectively, were established in the ipsilateral hind paw 2 days after CCI and lasted for at least 14 days after surgery (Fig. 2A–B). The contralateral hind paw did not show significant changes in paw withdrawal threshold or cumulative scores (data not shown). Rats were monitored during the 14 days after surgery, and no unexpected side effects were observed.

### Effect of Nicorandil on Mechanical and Cold Allodynia in CCI Rats

**Fig. 3:**
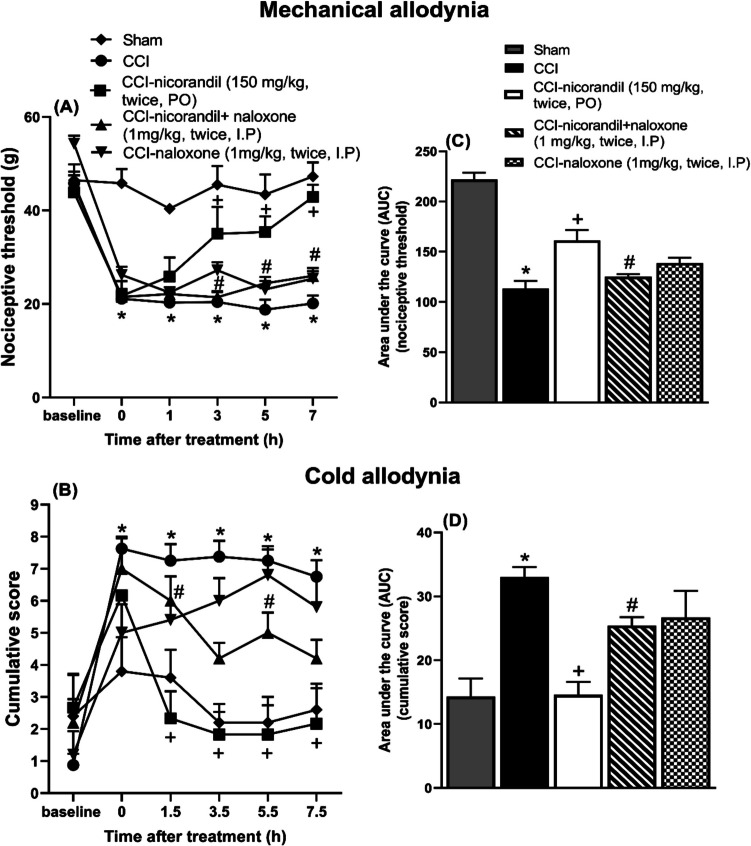
Effect of nicorandil (150 mg/kg, twice, 2 h apart, PO) on the nociceptive threshold (**A**), the cumulative score (**B**), and the corresponding area under the curves (**C** and **D**) assessed by the von Frey and acetone test, respectively, in chronic constriction injury (CCI) rats. Naloxone (1 mg/kg, twice, 2 h apart, I.P) was used as a standard opioid blocker in nicorandil-treated rats and was administered simultaneously with each dose of nicorandil. All results were expressed as mean ± SEM (*n* = 5–8). Results were analyzed by two-way ANOVA in temporal course figures (**A**–**B**) and one-way ANOVA in AUC figures (**C**–**D**) followed by Tukey’s post hoc test. *P < 0.05 vs sham; + *P* < 0.05 vs CCI; #*P* < 0.05 vs CCI-nicorandil

Nicorandil (150 mg/kg, twice, 2 h apart, PO) significantly attenuated the mechanical allodynia of CCI rats at 3, 5, and 7 h post-injection (*P* < 0.05) (Fig. 3A). In addition, nicorandil significantly ameliorated cold allodynia at all time intervals (*P* < 0.05) (Fig. 3B). Calculated AUC data also confirmed the analgesic effect of nicorandil (*P* < 0.05) (Fig. 3C and D). Prior administration of naloxone (1 mg/kg, twice, 2 h apart, I.P) partially reversed the antinociceptive activity of nicorandil at 3, 5, and 7 h in mechanical allodynia (Fig. 3A) and at 1.5 and 5.5 h in the cold allodynia test (*P* < 0.05) (Fig. 3B) which was confirmed by the calculated AUC data (*P* < 0.05) (Fig. 3C and D). Nicorandil showed higher % inhibition of mechanical and cold allodynia compared to the standard drug gabapentin (42.15% vs 36.14% and 55.98% vs 37.35%, respectively), which is used in the treatment of neuropathic pain (data not shown).

### Effect of Nicorandil on Licking Time and Number of Flinches Induced by Formalin

**Fig. 4:**
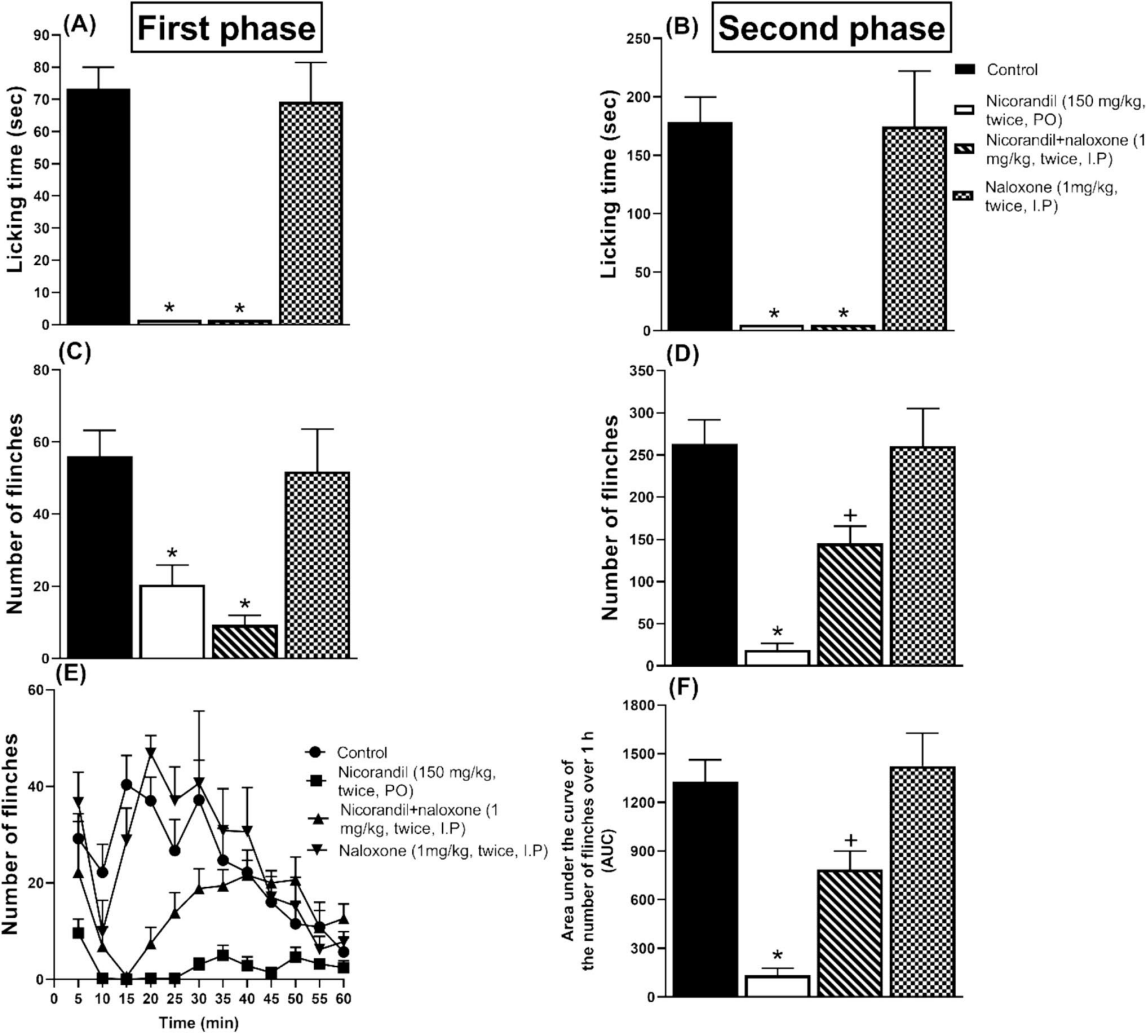
Effect of nicorandil (150 mg/kg, twice, 2 h apart, PO) in absence and presence of naloxone (1 mg/kg, twice, 2 h apart, I.P) on licking time (**A**, **B**), number of flinches (**C**, **D**) induced by ipl injection of formalin (50 µl, 1%) measured in first and second phases, number of flinches over 1 h (**E**) and total area under the curve for flinches (**F**). All results were expressed as mean ± SEM (*n* = 5–8). Results were analyzed by one-way ANOVA followed by Tukey’s post hoc test; *P < 0.05 vs control; + *P* < 0.05 vs nicorandil

Ipl injection of formalin induced nociceptive response as assessed by licking and flinches of the injected paw during both phases of the formalin test. Compared to the control group, nicorandil (150 mg/kg, twice, 2 h apart, PO) showed significant analgesic activity as observed by the reduction in licking time and number of flinches in both the first (Fig. 4A and C) and second (Fig. 4B and D) phases of the formalin test (*P* < 0.05). Pre-treatment with naloxone (1 mg/kg, twice, 2 h apart, I.P) blocked the effect of nicorandil on the flinches’ response in the second phase of the formalin test (*P* < 0.05) (Fig. 4D). The time course of the total number of flinches over 1 h was illustrated in Fig. 4E. Naloxone partially reversed the falls noted in the calculated AUC of the total number of flinches of the formalin test (*P* < 0.05) (Fig. 4F).

### Correlation Between Licking Time, Number of Flinches in Formalin Test, and Cold Allodynia Induced by CCI

**Fig. 5:**
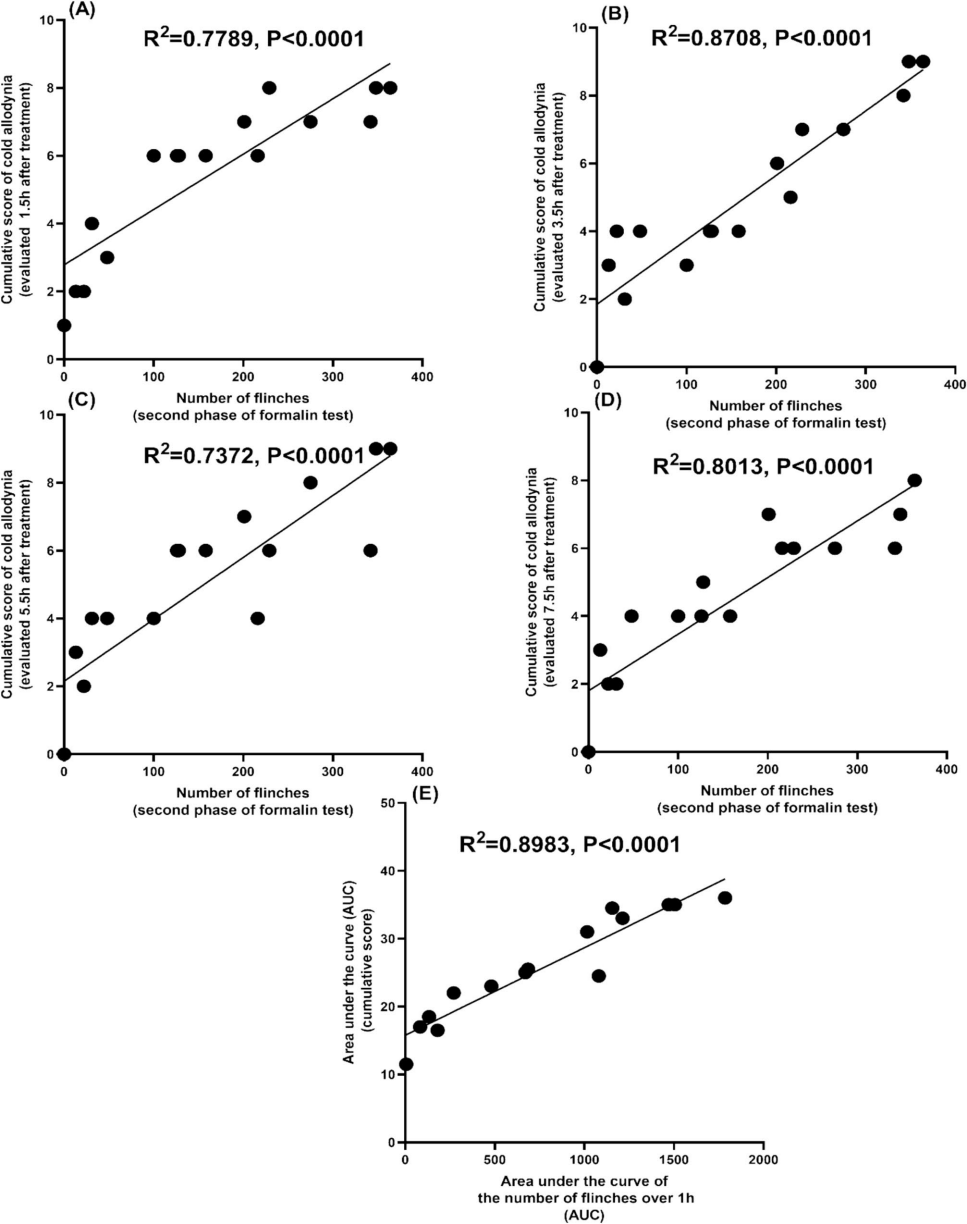
Pearson correlation between the number of flinches of the second phase of the formalin test and cold allodynia induced by CCI in rats evaluated at 1.5, 3.5, 5.5, and 7.5 h (**A**, **B**, **C**, and **D**, respectively), after treatment with vehicle (CMC), nicorandil (150 mg/kg, twice, 2 h apart, PO) and nicorandil + naloxone (1 mg/kg, twice, 2 h apart, I.P) and between AUC of the number of flinches over 1 h and AUC of cumulative score in the cold allodynia test (**E**)

Pearson correlation analysis indicated a moderate and strong positive association between the number of flinches of second phase of the formalin test and the cumulative score of the cold allodynia test evaluated at 1.5 h (Fig. 5A), 3.5 h (Fig. 5B), 5.5 h (Fig. 5C), and 7.5 h (Fig. 5D) (*R*^2^ = 0.7789, *P* < 0.0001, *R*^2^ = 0.8708, *P* < 0.0001, *R*^2^ = 0.7372, *P* < 0.0001, *R*^2^ = 0.8013, *P* < 0.0001), respectively, after vehicle (CMC) or treatment with nicorandil (150 mg/kg, twice, 2 h apart, PO), nicorandil + naloxone (1 mg/kg, twice, 2 h apart, I.P). Additionally, AUC of the total number of flinches over 1 h showed a strong positive correlation with the corresponding AUC of the cumulative score of the cold allodynia test in CCI rats (Fig. 5E) (*R*^2^ = 0.8983, *P* < 0.0001).

**Fig. 6:**
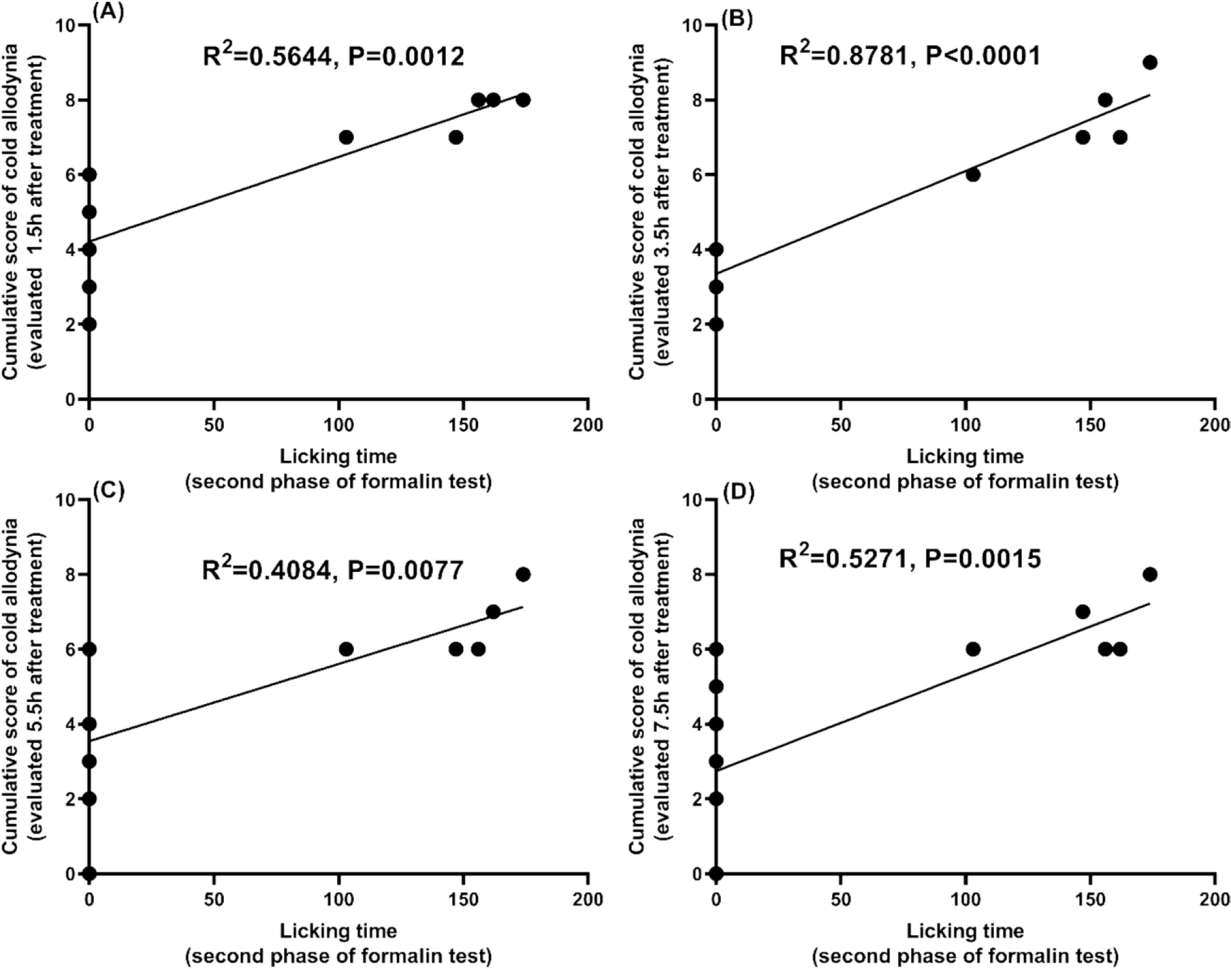
Pearson correlation between licking time of the second phase of the formalin test and the cumulative score of cold allodynia induced by CCI in rats evaluated at 1.5, 3.5, 5.5, and 7.5 h (**A**, **B**, **C**, and **D**, respectively), after treatment with vehicle (CMC), nicorandil (150 mg/kg, twice, 2 h apart, PO) and nicorandil + naloxone (1 mg/kg, twice, 2 h apart, I.P)

There is a weak positive correlation (Fig. 6A, C, and D) and a strong positive correlation (Fig. 6B) between licking time of second phase of the formalin test and the cumulative score of the cold allodynia test evaluated at 1.5 h (Fig. 6A), 3.5 h (Fig. 6B), 5.5 h (Fig. 6C) and 7.5 h (Fig. 6D) (*R*^2^ = 0.5644, *P* = 0.0012, *R*^2^ = 0.8781, *P* < 0.0001, *R*^2^ = 0.4084, *P* = 0.0077, *R*^2^ = 0.5271, *P* = 0.0015), respectively, after vehicle (CMC) or treatment with nicorandil (150 mg/kg, twice, 2 h apart, PO), nicorandil + naloxone (1 mg/kg, twice, 2 h apart, I.P).

### Effect of Nicorandil on Licking Time and Number of Flinches Induced by Capsaicin

**Fig. 7:**
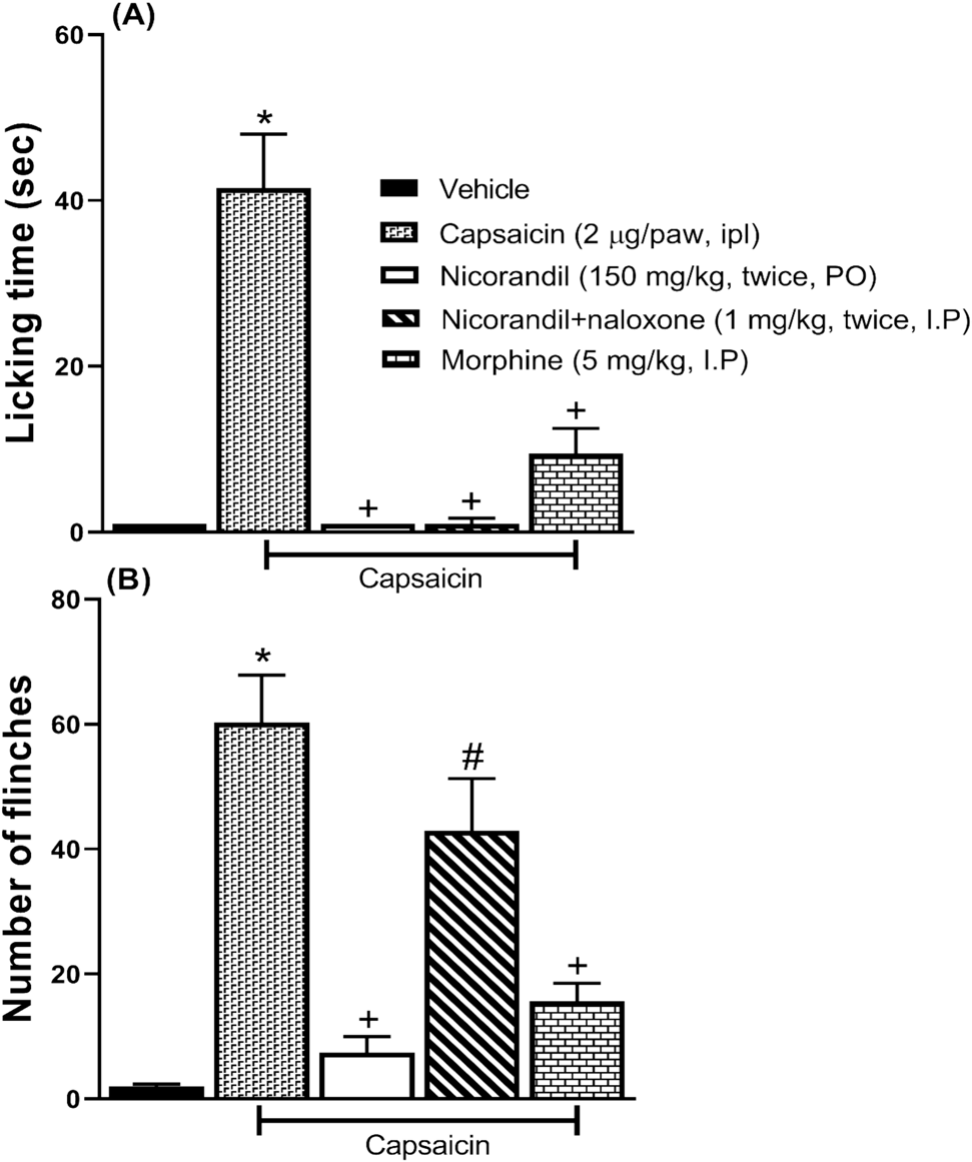
Effect of nicorandil (150 mg/kg, twice, 2 h apart, PO) and morphine (5 mg/kg, I.P) on licking time (**A**) and number of flinches (**B**) induced by ipl injection of capsaicin (2 µg/paw). Naloxone (1 mg/kg, twice, 2 h apart, I.P) was used as a standard opioid blocker in nicorandil-treated rats. All results were expressed as mean ± SEM (*n* = 5–8). Results were analyzed by one-way ANOVA followed by Tukey’s post hoc test; *P < 0.05 VS capsaicin vehicle; + *P* < 0.05 vs capsaicin, # *P* < 0.05 vs nicorandil

Capsaicin (2 µg/paw, ipl) caused a nociceptive response characterized by licking (Fig. 7A) and flinches (Fig. 7B) of the paw. Nicorandil (150 mg/kg, twice, 2 h apart, PO) significantly reduced the nociceptive response (*P* < 0.05). Pre-treatment with naloxone (1 mg/kg, twice, 2 h apart, I.P) attenuated the effect of nicorandil on the flinches’ response (*P* < 0.05). Morphine (5 mg/kg, I.P) significantly reduced both the licking time and the number of flinches (*P* < 0.05). Nicorandil showed higher % inhibition of both licking time and the number of flinches compared to morphine (100% VS 77.10% and 87.15% vs 74%), respectively.

### Effect of Nicorandil on Number of Writhes Induced by Acetic Acid

**Fig. 8:**
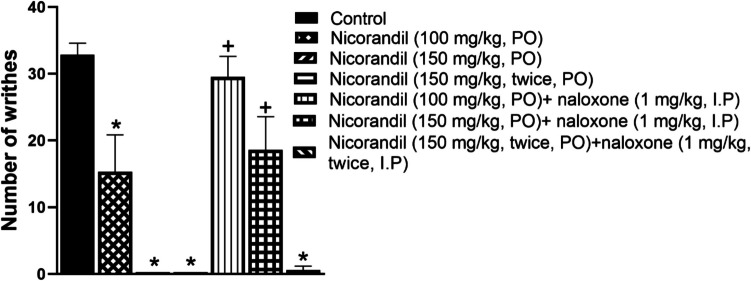
Effect of oral nicorandil (100, 150, 150 mg/kg, twice, 2 h apart, PO) alone and in presence of naloxone (1 mg/kg, twice, 2 h apart, I.P) on number of writhes induced by acetic acid (0.6%, 10 ml/kg, I.P). All results were expressed as mean ± SEM (*n* = 5–8). Results were analyzed by one-way ANOVA followed by Tukey’s post hoc test. *P < 0.05 vs control; + *P* < 0.05 vs nicorandil-treated groups

As presented in Fig. 8, I.P administration of acetic acid caused a writhing response in rats. Oral nicorandil (100 mg/kg, 150 mg/kg, 150 mg/kg, twice, 2 h apart) significantly decreased the number of writhes with % inhibition 53.36%, 100%, and 100%, respectively. Naloxone blocked the antinociceptive action of nicorandil (*P* < 0.05).

**Fig. 9:**
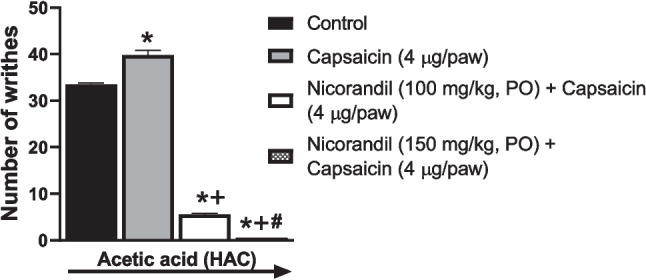
Effect of oral nicorandil (100, 150 mg/kg) in the presence of capsaicin (4 µg/paw, ipl) on the number of writhes induced by acetic acid (0.6%, 10 ml/kg, I.P). All results were expressed as mean ± SEM (*n* = 3–4). Results were analyzed by one-way ANOVA followed by Tukey’s post hoc test. *P < 0.05 vs control acetic acid; + *P* < 0.05 vs acetic acid + capsaicin; #*P* < 0.05 vs nicorandil 100 mg/kg

To further verify the effect of nicorandil on TRPV1 receptors, combined nicorandil (100, 150 mg/kg, PO) and capsaicin was found to abolish acetic acid-induced writhing response (Fig. 9). The inhibitory effect of nicorandil on the high concentration of the TRPV1 agonist, capsaicin (4 µg/paw) proved the pharmacological interaction between nicorandil and TRPV1 receptors, providing functional evidence that nicorandil’s antinociception is TRPV1-dependent.

### Effect of Intraplantar Nicorandil on Nociceptive Tests

**Fig. 10:**
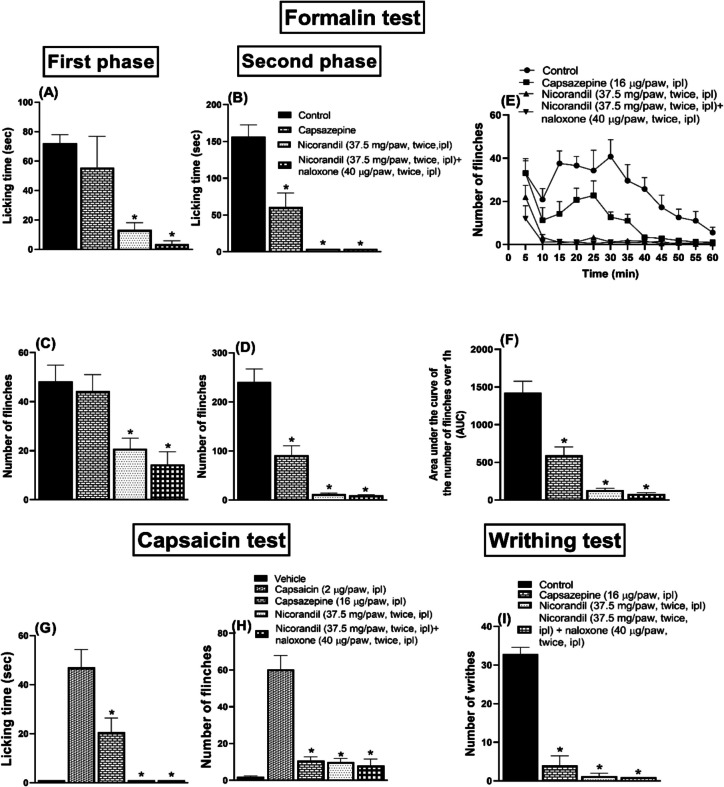
Effect of intraplantar injection of nicorandil (37.5 mg/paw, twice, 2 h interval) and intraplantar injection of capsazepine (16 µg/paw) on licking time (**A**–**B**), number of flinches (**C**–**D**) of first and second phases, respectively, total number of flinches over 1 h (**E**) and area under the curve of flinches (**F**) induced by formalin injection (50 µl, 1%) and licking time (**G**) and number of flinches (**H**) induced by capsaicin test (2 µg/paw) and number of writhes (**I**) induced by acetic acid injection (0.6%, 10 ml/kg, I.P). Naloxone (40 µg/paw, twice, ipl) was administered as a standard opioid antagonist. All results were expressed as mean ± SEM (*n* = 5–8). Results were analyzed by one-way ANOVA followed by Tukey’s post hoc test. *P < 0.05 vs control or capsaicin

As depicted in Fig. 10, intraplantar nicorandil (37.5 mg/paw, twice, 2 h apart) significantly reduced licking time (Fig. 10A and B), the number of flinches (Fig. 10C and D) in both phases of formalin and the AUC of flinches response in the formalin test (*P* < 0.05) (Fig. 10F). Capsazepine significantly attenuated licking time and the number of flinches of the second phase of the formalin test and the AUC of flinches response (*P* < 0.05) (Fig. 10B, D, and F). The time course of the number of flinches over 1 h was shown in Fig. 10E. Naloxone (40 µg/paw, twice, 2 h apart, ipl) did not reverse nicorandil’s antinociception mentioned before.

Capsaicin (2 µg/paw, ipl) showed licking (Fig. 10G) and flinches response (Fig. 10H) compared to the vehicle (*P* < 0.05). Intraplanar nicorandil (37.5 mg/paw, twice, 2 h apart) significantly reduced the licking time and the number of flinches (*P* < 0.05). Similar % inhibition of flinches’ response was recorded for both nicorandil and capsazepine (16 µg/paw) 84.78% and 82.33%, respectively. Nicorandil showed higher % inhibition of licking time compared to capsazepine (100%, 56.17%, respectively). Pre-treatment with naloxone (40 µg/paw, twice, 2 h apart, ipl) did not change the responses of ipl nicorandil (*P* > 0.05).

As shown in Fig. 10I, ipl administration of both nicorandil (37.5 mg/paw, twice, 2 h apart) and capsazepine (16 µg/paw) significantly reduced the number of acetic acid-induced writhing (*P* < 0.05). ipl nicorandil showed a higher % inhibition of the writhing response compared to ipl capsazepine (96.19%, 87.83%, respectively). ipl administration of naloxone (40 µg/paw, twice, 2 h apart) did not reverse the effect of ipl nicorandil in the writhing test (*P* > 0.05).

Table 1 shows the comparison between the effect of systemic nicorandil (150 mg/kg, twice, 2 h apart, PO), intraplantar nicorandil (37.5 mg/paw, twice, 2 h interval) and capsazepine (16 µg/paw) on the nociceptive responses induced by formalin (50 µl, 1%, ipl), capsaicin (2 µg/paw, 20 µl, ipl), and acetic acid (0.6%, 10 ml/kg, I.P).

**Table 1:** Comparison between the effect of systemic nicorandil (150 mg/kg, twice, 2 h apart, PO), intraplantar nicorandil (37.5 mg/paw, twice, 2 h interval), and capsazepine (16 µg/paw) on the nociceptive responses induced by formalin (50 µl, 1%, ipl), capsaicin (2 µg/paw, 20 µl, ipl), and acetic acid (0.6%, 10 ml/kg, I.P)

Test		Systemic nicorandil (150 mg/kg, twice, 2 h apart, PO)	Ipl nicorandil (37.5 mg/paw, twice, 2 h interval)	Ipl capsazepine (16 µg/paw)
% Inhibition				
Formalin test (50 µl, 1%)				
1 st phase	Licking time	100%	81.72%	22.77%
	Number of flinches	63.47%	56.89%	8.22%
2nd phase	Licking time	100%	100%	61%
	Number of flinches	92.77%	94.94%	61.88%
Capsaicin test (2 µg/paw)				
Licking time		100%	100%	56.17%
Number of flinches		87.15%	84.78%	82.33%
Acetic acid test (0.6%, 10 ml/kg)				
Writhing		100%	96.19%	87.83%

### Effect of Nicorandil on TRPV1 Expression Level in DRG

**Fig. 11:**
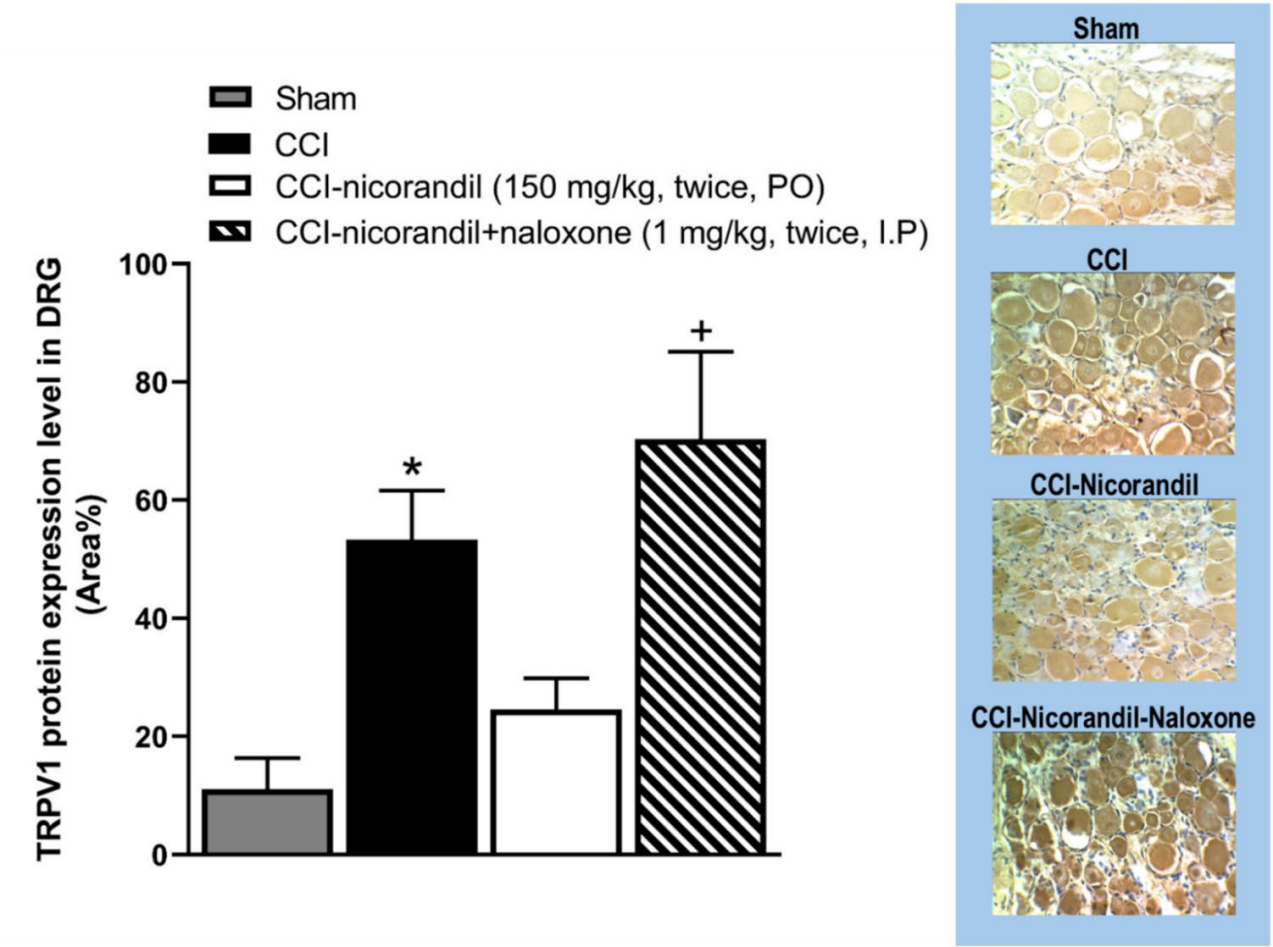
Effect of nicorandil (150 mg/kg, twice, 2 h apart, PO) on immunohistochemical protein expression of TRPV1 channels in DRG of CCI rats. Naloxone (1 mg/kg, twice, 2 h apart, I.P) was used as a standard opioid blocker in nicorandil-treated rats. Representative images for immuno-stained sections are also shown. All results were expressed as mean ± SEM (*n* = 5). Results were analyzed by one-way ANOVA followed by Tukey’s post hoc test; *P < 0.05 vs sham; + *P* < 0.05 vs CCI-nicorandil

As illustrated in Fig. 11, immunohistochemical data showed that the protein expression of TRPV1 channels was elevated in CCI rats compared to sham counterparts (*P* < 0.05). Intriguingly, CCI failed to elevate the TRPV1 expression in nicorandil (150 mg/kg, twice, 2 h apart, PO)-treated rats. Moreover, naloxone (1 mg/kg, twice, 2 h apart, I.P) cotreatment reversed the nicorandil-related falls in TRPV1 expression levels (*P* < 0.05).

### Molecular Docking Study

**Fig. 12:**
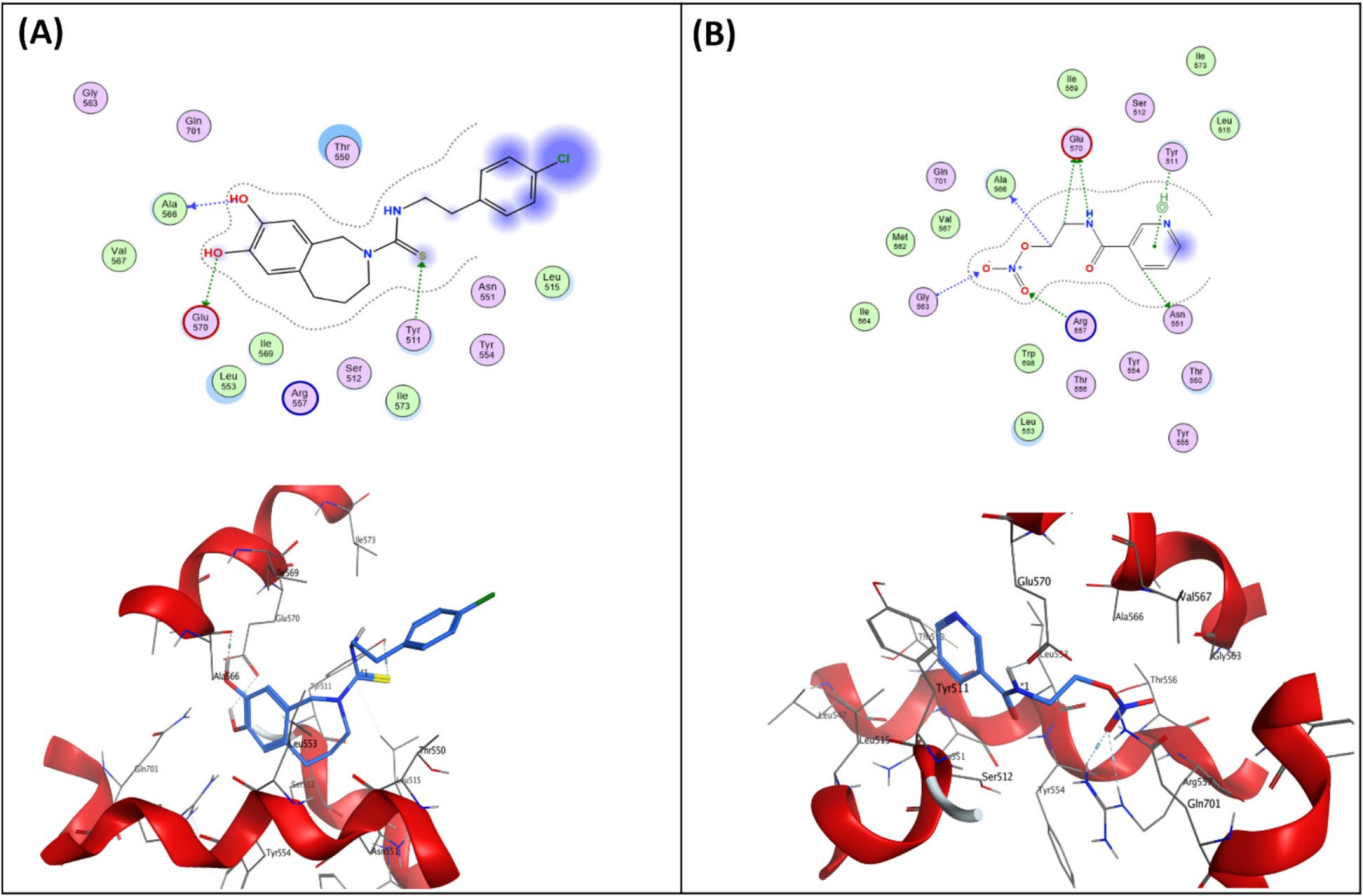
Docking and binding pattern of (**A**) capsazepine and (**B**) nicorandil into TRPV1 active site (PDB ID: 8GFA) in 2D (upper panels) and 3D (lower panels)

Relative positioning of nicorandil with respect to capsazepine, hydrogen bonds, and docking scores was used to assess its binding affinity to the TRPV1 receptor. The docked pose of capsazepine into the TRPV1 active site displayed three hydrogen bonds with Tyr511, Ala566, and Glu570 residues (Fig. 12A).

Nicorandil was well anchored into the TRPV1 active site, showing a better docking score and molecular interactions than capsazepine (Table 2). Nicorandil reserved the three key interactions similar to capsazepine. It established two hydrogen bonds with Ala566 and Glu570, together with a pi-hydrogen contact with TYR511 (Fig. 12B).

**Table 2:** Docking scores of capsazepine and nicorandil against TRPV1 receptor

Compound	Docking scores (Kcal/mol)
	TRPV1
Capsazepine	− 5.7264
Nicorandil	− 6.3747

### Effect of Nicorandil on the Histopathological Changes Induced by CCI

**Fig. 13:**
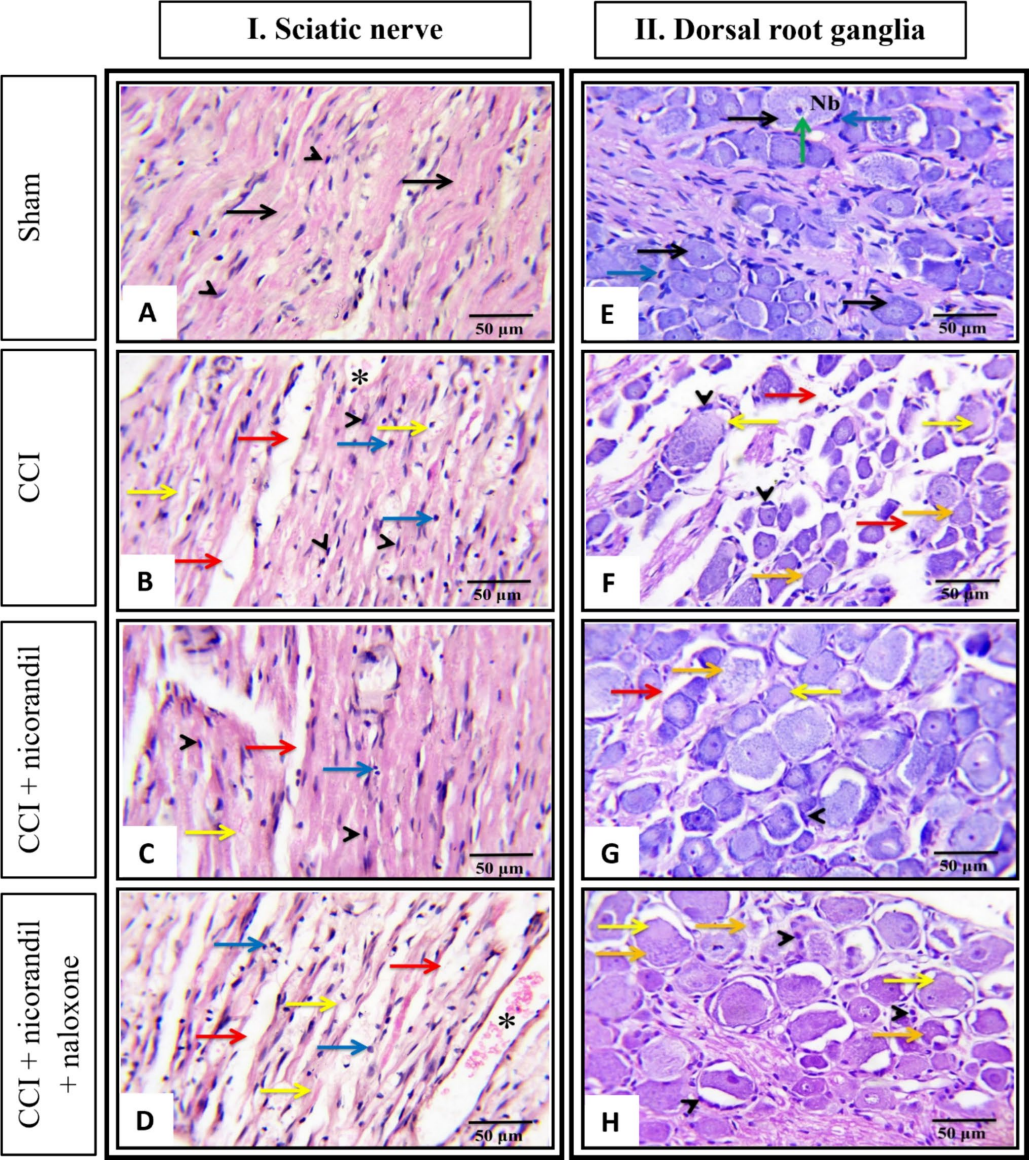
Effect of nicorandil (150 mg/kg, twice, 2 h apart, PO) on CCI-induced histopathological changes in longitudinal sections of sciatic nerve (**A**–**D**) and transverse section of dorsal root ganglia (**E**–**H**) of sham (**A** and **E**), CCI (**B** and **F**), nicorandil (150 mg/kg, twice, 2 h apart, PO)-treated CCI rats (**C** and **G**) and naloxone (1 mg/kg, twice, 2 h apart, I.P) pretreated CCI rats (**D** and **H**), respectively. All images were captured under magnification power × 400 (scale bar, 50 µm) (*n* = 5). Sciatic nerve: normal nerve fibers (black arrow), myelin sheets degeneration (yellow arrow), areas of edema (red arrow), Schwann cells nuclei (arrow heads), mononuclear cells infiltration (blue arrow), and increase in capillary size (*). Dorsal root ganglia: normal nerve cells (black arrow), Nissl body in the cytoplasm of nerve cells (Nb), satellite cells (blue arrow), nucleus with prominent nucleolus (green arrow), degenerated neurons with absence of Nissl bodies (yellow arrow), areas of edema (red arrow), hypertrophy of satellite cells (arrow heads), and the neuronal cells showed loss of the nuclei and showed a ghost cell-like morphology (orange arrow)

Microscopic examination of hematoxylin and eosin (H&E) stained longitudinal sections of the sciatic nerve tissues obtained from the different studied groups is shown in Fig. 13I and A–D. Sciatic nerves of the control rats (Sham) showed a clear organization of nerve fibers, myelin sheaths, and number of Schwann cell nuclei with absence of infiltrating cells (Fig. 13A). Conversely, longitudinal sections of the sciatic nerve of CCI rats showed general loss of the nerve fibers architecture, Wallerian degeneration of sciatic axons with presence of degenerated myelin sheath associated with inflammatory cells infiltration and increased number of fibroblasts and Schwann cells. Also, an increase in the size of capillaries associated with edematous surrounding areas was noticed (Fig. 13B). Notably, in the nicorandil group, partial restoration of the normal architecture of the nerve fibers was achieved, as most nerve fibers were intact with only slight separation between them (Fig. 13C). Inversely, concurrent administration of naloxone partially attenuated the drug’s impact (Fig. 13D).

Microscopic examination of H&E-stained transverse sections of DRG tissues obtained from the different studied groups is shown in Fig. 13II and E–H). In the sham group, the DRGs show normal histological structure. The ganglion cells had a large cell body with abundant cytoplasm and an oval nucleus with a prominent nucleolus, and abundant Nissl bodies in the cytoplasm of nerve cells were observed. Additionally, the cells were surrounded by thin, flattened satellite cells (Fig. 13E). However, in the CCI rats DRG, degenerated nerve cells were found, as indicated by the disappearance of Nissl bodies. Other neuronal cells demonstrated loss of nucleoli with a ghost cell-like morphology (Fig. 13F). The recovery of nerve cell morphology was dramatically observed in the nicorandil-treated group. Moreover, Nissl bodies and nucleoli reappeared with nicorandil treatment (Fig. 13G). Contrarily, concurrent administration of naloxone partially attenuated the nicorandil effect, showing an increase in satellite cell hypertrophy, neuronal degeneration, and ghost-like morphology cells (Fig. 13H).

## Discussion

The current study presents novel findings on the implication of TRPV1/opioid receptors in the antinociceptive activity of nicorandil in neuropathic pain induced by CCI and nociceptive pain models, namely, formalin, capsaicin, and writhing tests. Systemic and intraplantar nicorandil induced similar antinociception in the nociceptive pain models (Table 1). Molecularly, nicorandil halted TRPV1 protein expression in CCI rats, which is confirmed by docking studies, as nicorandil interacts with TRPV1 receptors and showed activity superior to the standard TRPV1 blocker, capsazepine. Moreover, nicorandil restored the histopathological damage induced by CCI in the sciatic nerve and DRG. The beneficial effects of nicorandil were blocked by the opioid receptor antagonist naloxone, thereby implicating the role of opioid signaling in addition to TRPV1 channels in nicorandil antinociception. That said, nicorandil antinociceptive potential in neuropathic pain is mediated through TRPV1/opioid signaling.

Neuropathic pain is a common chronic pain condition caused by a lesion or disease of the somatosensory nervous system with major impact on quality of life, yet its pharmacotherapeutic management is still an unmet medical need [3]. Elicited pain may spread to adjoining areas causing peripheral and central sensitization of nociceptive pathways. Thereby, the multifaceted pathogenesis of neuropathic pain includes alteration in ion channels such as K_ATP_, sodium, and calcium channels [5]. Most importantly, TRPV1 channels upregulation after nerve injury and alteration in the opioid pathway are also implicated in neuropathic pain [38, 39].

Nicorandil is an anti-anginal agent, which has been recommended as a second-line treatment for chronic stable angina based on its (K_ATP_) channel opener activity and donation of nitric oxide (NO) [16]. In the present study, nicorandil mediated a potent antinociceptive effect in four pain models sharing a common nociceptive target, which are TRPV1 channels and full/partial characteristics of neuropathic pain. Herein, nicorandil attenuated mechanical and cold allodynia induced by CCI, a pain model that causes peripheral nerve injury and is widely conducted to study neuropathic pain [40]. Similar to human neuropathic pain, CCI causes an inflammatory response with absolute and partial damage of A and C fibers, respectively [25, 41]. Previous reports showed that nicorandil mitigated the neuropathic pain induced by paclitaxel and ameliorated the mechanical allodynia noted in CCI rats [20] as well as postsurgical chronic pain [19].

The crucial role of ion channels in the development of neuropathic pain has been previously illustrated [42]. Particularly, nerve injury caused downregulation of ATP-sensitive potassium channel (K_ATP_) currents in DRG [43]. Favorably, potassium channel openers have demonstrated analgesic activity in neuropathic pain in animals [44]. One could argue that the antinociceptive effect of nicorandil is mediated via K_ATP_ channels. The nicorandil antinociceptive effect in the formalin test was not blocked by glibenclamide [17]. Moreover, a recent report showed that glibenclamide did not block the antinociceptive effect of nicorandil/morphine combination against tail flick and formalin test in liver fibrotic rats [22]. However, findings were expanded to clarify that the antinociceptive activity of nicorandil may not be principally attributed to K_ATP_ channel opening and blocking of ATP-dependent potassium channels does not seem to play a role in the nicorandil antiallodynic response. This notion was supported by the experimental finding (behavioral data from a parallel unpublished dataset) showing that co-administration of glibenclamide, a selective K_ATP_ channel blocker, did not alter the antinociceptive effects of nicorandil on either mechanical or cold allodynia in CCI rats nor in the formalin-induced inflammatory pain model. These results suggest that nicorandil’s analgesic effects are not primarily mediated via the K_ATP_ channels.

The present study brought up three novel evidences that reinforce the action of nicorandil on TRPV1 channels. First, the current molecular docking data of nicorandil into the TRPV1 active site (PDB ID: 8GFA) showed an intriguing binding profile with affinity superior to capsazepine, which is consistent with the higher percent inhibition of licking time in the capsaicin test by nicorandil compared to capsazepine (100% vs 56.17%) as shown in our results. Second, the immunohistochemical analysis of DRG isolated from CCI rats demonstrated that the upregulation of the expression of TRPV1 receptors in CCI rats was halted by nicorandil and reversed by naloxone. TRPV1 channels seem to play a crucial role in neuropathic pain. Of particular note, the upregulation of TRPV1 channels in DRG after nerve injury is reported to trigger pain [39]. Alteration of TRPV1 expression is reported in different models of neuropathy [9–11, 45]. Silencing of the spinal TRPV1 gene is found to alleviate neuropathic pain in the rat CCI model [46]. On the other hand, increased TRPV1 expressions might reflect peripheral sensitization. However, the observed reversal of CCI-induced TRPV1 upregulation in DRG by nicorandil and the restoration of this upregulation by the tested blocker naloxone support a modulatory role of nicorandil on TRPV1 at the protein level, beyond nonspecific downregulation secondary to analgesia. A recent reference indicates the orofacial antinociceptive activity of nicorandil in adult zebrafish and highlights the potential of nicorandil as an analgesic through its interaction with TRPV1 receptors employing behavioral and molecular docking studies [47].

Lastly, the current behavioral experiments strengthen the conclusion that nicorandil antinociception is directly related to TRPV1 blockade, as supported by inhibition of the capsaicin response in acetic acid-induced writhing. Amazingly, nicorandil was found to inhibit the nociceptive response induced by capsaicin, a potent agonist of TRPV1 receptors. Moreover, TRPV1 pharmacological inhibition attenuated neuropathic pain in different experimental studies [10, 12].

The current findings indicated that nicorandil demonstrated antinociceptive activity in both phases of the formalin test. Interestingly, systemic and ipl injection of nicorandil abolished formalin-induced nociceptive response. Formalin-evoked nociception originated from tissue damage-triggered activation of TRPV1, TRPA1, and subsequent inflammatory cascade [17]. This finding tempted us to investigate the effect of capsazepine, a TRPV1 antagonist, on formalin-induced pain. Herein, capsazepine decreased the number of flinches and the licking response in the second phase of the formalin test. TRPV1 antagonists are reported to decrease the number of flinches caused by subcutaneous injection of formalin [48]. Local application of capsazepine inhibited paw licking time in both phases of the formalin test [49]. These findings suggest the implication of TRPV1 channels in formalin-evoked pain. TRPV1 channels are also implicated in mediating the secondary allodynia and hyperalgesia induced by formalin, as evidenced by increased expression of TRPV1 in DRG following formalin administration [50]. Presumably, if nicorandil follows the same scenario, our findings suggest that the antinociceptive effect of nicorandil in the formalin test is mediated partially through TRPV1 channels.

Formalin causes neuropathic pain-like characteristics presented as long-lasting mechanical hyperalgesia and allodynia. Although these effects were observed with a higher concentration of formalin than that employed in our design [51], the noted antinociceptive activity of nicorandil in the formalin test may suggest its promising potential against neuropathic pain characteristics. Nicorandil demonstrated antinociceptive activity during both phases of the formalin test and previously showed potential antinociception in mice [16, 17]. In agreement with the aforementioned data, here nicorandil revealed antinociceptive activity during both phases of the formalin test, suggesting both anti-inflammatory and analgesic potentials.

A subsidiary but crucial finding is the establishment of pharmacological correlation between the CCI model and formalin test. The current experimental data revealed a positive linear correlation between the cumulative score of cold allodynia and the number of flinches in the second phase of the formalin test using the Pearson correlation analysis. Remarkably, this was observed after 3.5 and 7.5 h of nicorandil administration in CCI rats. The second phase of the formalin test and the CCI cold allodynia test are related, as several analgesic compounds demonstrated comparable results in both experiments, including morphine, fentanyl, and flunarizine [52] supporting the validity of formalin testing for neuropathic pain.

Further, the present findings indicated that nicorandil mediated antinociception against capsaicin and acid induced pain, which are mainly related to TRPV1 activation. Intraplantar capsaicin causes a nociceptive response due to TRPV1 stimulation [53]. The involvement of TRPV1 channels in acetic acid writhing pain has been reported. For instance, capsazepine, a well-known TRPV1 antagonist, reduced the acetic acid-induced writhing response [54]. Additionally, destruction of capsaicin-sensitive neurons in mice neonates showed a reduced writhing response, indicating a crucial role of TRPV1 channels in acetic acid pain [54]. Moreover, resiniferatoxin, an ultrapotent TRPV1 agonist causing full desensitization and ablation of sensory neurons [55] decreased acetic acid-induced pain in mice [56].

Nicorandil mediated antinociceptive effect in CCI rat model, formalin, capsaicin and writhing tests, was blocked by the opioid receptor antagonist, naloxone. This finding was supported by current immunohistochemical analysis and histological examination that naloxone showed partial reversal of nicorandil effect on TRPV1 expression and structural damage of DRG and sciatic nerve, respectively, in CCI rats. Recent reports point to the role of opioid receptors in mediating nicorandil analgesic activity [21, 22]. Nicorandil antinociceptive effect is reported to be attenuated by naltrexone in paclitaxel-induced neuropathic pain [20]. Supporting this notion, nicorandil exhibited activity in models of inflammatory pain induced by ipl injection of carrageenan and nociceptive pain induced by exposure to noxious heat via opioid pathways activation [18]. The demonstration that the antinociceptive effect induced by nicorandil is markedly attenuated by opioid antagonists provides solid information about the involvement of opioid signaling in mediating the activity of this antianginal drug.

Co-expression of TRPV1 and µ-opioid receptors does exist in DRG neurons, and their cross talk is plausible. Thus, a dual acting drug targeting both TRPV1 channels and opioid receptors would be of great value in pain management. Recently, nicorandil was found to interact with opioid receptors, with the highest selectivity for delta opioid receptors compared to morphine [22]. Additionally, the favorable binding of nicorandil on TRPV1 channels has great value in the management of neuropathic pain. Here, naloxone reversed nicorandil effects on TRPV1 expression and its analgesic potential against capsaicin and acetic acid writhing tests. Our results suggested that nicorandil targeted both TRPV1 and opioid receptors. Nicorandil, therefore, acts as a bifunctional drug, providing the pain relief benefits of opioid agonists while avoiding their side effects through its TRPV1 antagonistic activity. This is the first finding to demonstrate the binding affinity of nicorandil to TRPV1 channels and report that the beneficial impact of nicorandil on neuropathic and nociceptive pain is partially mediated via the TRPV1/opioidergic signaling pathway.

Multiple factors such as chemotherapy and inflammation can trigger alteration in TRPV1 receptors in the dorsal root ganglion. Reportedly, acute treatments involving pain or nerve injury increased expression, membrane insertion, and functional sensitization of TRPV1 receptors, leading to pain hypersensitivity. Supporting our behavioral assessment using the acetone spray test, 4 days of treatment with oxaliplatin increased acute cold hypersensitivity and induced changes in expression of transient receptor potential channels in the dorsal root ganglion in rats [57]. Chemokine CCL2 and its receptor expression increased in DRG as early as 4 h after a single dose treatment of oxaliplatin [58]. In the myocardial ischemia reperfusion model, TRPV1 expression increased in DRG tissue after 30 min of occlusion of the coronary artery and 2 h of reperfusion. Both protein and phosphorylation levels of TRPV1 and the immunofluorescence intensity of TRPV1 protein expression and channel activity of TRPV1 in DRG induced by cardiac ischemia have a critical role in heart injury. Intrathecal injection of the TRPV1 antagonist resiniferatoxin 10 min before the model decreased TRPV1 expression in DRG tissues [59]. Finally, acute injection of bortezomib induced neuropathic pain and increased TRPV1 expression in DRG and spinal cord measured by western blotting 1 h after injection [60]. In the same context, in the current study, the chronic constriction injury was done and animals were tested 14 days post-surgery, which is considered long enough to induce sensitization of TRPV1 in dorsal root ganglia and verify the antinociceptive response induced by acute doses of nicorandil.

Such privileged effect of nicorandil in suppressing neuropathic and nociceptive pain appears to be reinforced by data obtained from histopathological studies. Signs of nerve damage, like the appearance of degenerated myelin sheath, increased number of fibroblasts, and inflammatory cells infiltration in the sciatic nerve were improved. Similarly, signs of disappearance of Nissl bodies in DRG were dramatically ameliorated by nicorandil and resulted in recovery of nerve architecture. Contrarily, prior exposure to naloxone partially abrogated the nicorandil effect, showing an increase in neuronal degeneration. These findings support the aforementioned advantageous effect of nicorandil on neuronal damage of neuropathic pain.

On the top of the present findings is the observation that both systemically and locally administered nicorandil showed comparable pain relief in capsaicin, formalin, and acetic acid writhing tests. Moreover, nicorandil demonstrated antinociceptive activity superior to that of the TRPV1 antagonist capsazepine (Fig. 10, Table 1). Surprisingly, opposite to the effect of systemic administration, the antinociceptive effect of ipl nicorandil in the nociceptive pain models was not mediated by an opioid mechanism since naloxone did not abolish nicorandil antinociception. The results of the effects of systemic and local administrations of drugs in different pain models are controversial. In one study, locally administered loperamide in the spinal nerve injury model showed enhancement of thermal and mechanical hyperalgesia, whereas it produced antinociception in naïve rats. However, systemically administered loperamide only enhanced heat and mechanical hyperalgesia [61]. In the formalin test, locally administered morphine showed superior effect compared to systemic administration [62]. In the CCI model, µ-opioid receptor agonists inhibited mechanical allodynia when administered intraplantarly with no significant effect upon systemic administration [63]. Magnesium chloride showed similar results when injected systemically and locally, relieving acute arthritis pain [64]. These findings enlighten that the peripheral analgesic potential of nicorandil can be mediated through peripheral TRPV1 and not mediated by an opioid mechanism.

Collectively, the data highlight that TRPV1/opioid signaling contributes to the therapeutic modality of nicorandil in neuropathic pain induced by CCI and nociceptive pain signals. The findings suggest that nicorandil could serve as an alternative option for managing neuropathic pain due to its multi-targeted mechanisms. Moreover, both systemic and local administration of nicorandil to rats revealed favorable analgesic effects. Together, the results emphasize that nicorandil should be further explored aiming at its readjusting in the treatment of various painful disorders.

## Data Availability

No datasets were generated or analysed during the current study.
